# Assessing the safety of bedaquiline: insight from adverse event reporting system analysis

**DOI:** 10.3389/fphar.2024.1382441

**Published:** 2024-05-09

**Authors:** Jiaqiang Wu, Hong Pan, Li Shen, Mingyi Zhao

**Affiliations:** ^1^ School of Life Sciences and Biopharmaceutical Science, Shenyang Pharmaceutical University, Shenyang, China; ^2^ Department of Pharmacy, Wuxi No.5 People’s Hospital, Wuxi, Jiangsu, China; ^3^ Department of Pharmacy, Suzhou Hospital, Affiliated Hospital of Medical School, Nanjing University, Suzhou, Jiangsu, China

**Keywords:** bedaquiline, adverse event, FAERS database, drug induced liver injury, network pharmacology

## Abstract

**Background:**

The development and marketing of Bedaquiline (BDQ) represent significant advancements in treating tuberculosis, particularly multidrug-resistant forms. However, comprehensive research into BDQ’s real-world safety remains limited.

**Purpose:**

We obtained BDQ related adverse event (AE) information from the US Food and Drug Administration’s Adverse Event Reporting System (FAERS) to assess its safety and inform drug usage.

**Methods:**

The AE data for BDQ from 2012 Q4 to 2023 Q3 was collected and standardized. Disproportionality analysis, including Reporting Odds Ratio (ROR), Proportional Reporting Ratio (PRR), Multi-item Gamma Poisson Shrinker (MGPS), and Bayesian Confidence Propagation Neural Network (BCPNN) was used to quantify signals of BDQ-related AEs. Logistic regression was used to analyze the individual data of hepatotoxicity and drug-induced liver injury, and multiple linear regression models were established. Additionally, network pharmacology was employed to identify the potential biological mechanisms of BDQ-induced liver injury.

**Results:**

We identified 2017 case reports directly related to BDQ. Our analysis identified 341 Preferred Terms (PTs) characterizing these AEs across 27 System Organ Classes (SOC). An important discovery was the identification of AEs associated with ear and labyrinth disorders, which had not been documented in the drug’s official leaflet before. Subgroup analysis revealed a negative correlation between BDQ-related liver injury and females (OR: 0.4, 95%CI: 0.3–0.6). In addition, via network pharmacology approach, a total of 76 potential targets for BDQ related liver injury were predicted, and 11 core target genes were selected based on the characterization of protein-protein interactions. The pathway linked to BDQ-induced liver injury was identified, and it was determined that the PI3K-Akt signaling pathway contained the highest number of associated genes.

**Conclusion:**

The analysis of the FAERS database revealed adverse events linked to BDQ, prompting the use of a network pharmacology approach to study the potential molecular mechanism of BDQ-induced liver injury. These findings emphasized the significance of drug safety and offered understanding into the mechanisms behind BDQ-induced liver injury. BDQ demonstrated distinct advantages, including reduced incidence of certain adverse events compared to traditional treatments such as injectable agents and second-line drugs. However, it is important to acknowledge the limitations of this analysis, including potential underreporting and confounding factors. This study provides valuable insights into the safety of BDQ and its role in the management of MDR-TB, emphasizing the need for continued surveillance and monitoring to ensure its safe and effective use.

## 1 Introduction

A significant global threat is posed by the development of drug-resistant tuberculosis (TB) (Mirzayev et al., 2021). In 2022, it is estimated that around 410,000 cases of multidrug-resistant/rifampicin-resistant (MDR/RR-TB) occurred worldwide, with nearly 17% of previously treated TB cases developing MDR/RR-TB (Organization WH, 2023).

Bedaquiline (BDQ), a novel diarylquinoline anti-tuberculosis (anti-TB) medication, brings hope for effectively treating multi-drug resistant tuberculosis (MDR-TB) (Zhu et al., 2023). Due to its unique mechanism of action in inhibiting mycobacterial ATP synthase, BDQ has demonstrated significant efficacy in controlling this formidable form of tuberculosis (Sarathy et al., 2019; Khoshnood et al., 2021). Although BQD treatment is effective, the increasing number of individuals targeted for it underscores the importance of a clinical assessment of its safety, particularly with the expanding indications for its use. It is crucial to comprehend the full spectrum of adverse events associated with BDQ in order to use it safely and effectively. It is believed that hepatotoxicity is one of the most severe adverse effects of classical anti-TB drugs such as isoniazid and rifampin (Saukkonen et al., 2006). The risk of hepatotoxicity associated with BDQ may limit its broad clinical utilization due to its side effects. A study of 1,162 patients in a prospective cohort, all on a BDQ regimen, revealed that around 16% of patients experienced hepatotoxicity (Gao et al., 2021). A retrospective cohort study based on the South Korea National Health Insurance System (NHIS) database found that a higher number of patients in the group receiving the BDQ regimen experienced liver injury compared to those treated with conventional therapy (Kim et al., 2023).

The FAERS database, the U.S. Food and Drug Administration’s Adverse Event Reporting System, serves as an open platform that is frequently utilized for the post-marketing safety evaluation of drugs (Jiang et al., 2024). It is a spontaneous reporting system that encompasses millions of drug adverse event (AE) reports, including all adverse event and medication use information (Zou et al., 2023). It provides a vast source of real-world data for the early identification of AEs (Zhao et al., 2023). AE information in the FAERS database is documented and analyzed individually, making the data more basic (Wang J. et al., 2023). By mining AE signals in FAERS, unrecognized safety signals can be managed during clinical development for risk management purposes (Wang F. et al., 2023). Network pharmacology is a discipline that utilizes systems biology and computer technology to construct networks and investigate the interactions between “disease-gene-target-drug” (Lin et al., 2022; Liu et al., 2023). It focuses on analyzing the molecular associations between drugs and therapeutic objects from a system-level perspective, revealing the systemic pharmacological mechanism of medications within the biological network (Liu et al., 2023).

**FIGURE 1:**
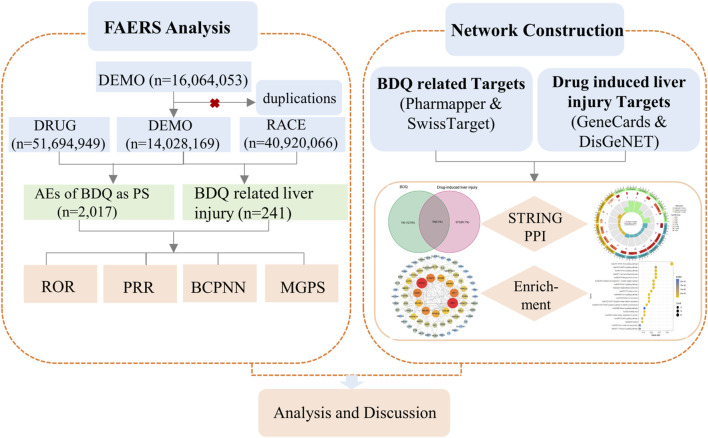
Flowchart of the study.

With the rise of artificial intelligence and the expansion of major public database platforms, data mining has proven to be a useful tool for uncovering indicators of adverse drug reactions. The purpose of this study was to identify the AEs associated with BDQ by analyzing the data from the FAERS database. Additionally, we explored the biological mechanism of BDQ-induced liver injury using network pharmacology to better understand its safety and improve the clinical application. The flowchart of the process was shown in Figure 1.

## 2 Materials and methods

### 2.1 Data source and collection

This study included BDQ ADE reporting data from the fourth quarter of 2012 (2012 Q4) to the third quarter of 2023 (2023 Q3). All ASCII data package information was available from the FAERS database (https://fis.fda.gov/extensions/FPD-QDE-FAERS/FPD-QDE-FAERS.html). The PubMed search used MeSH terms with the keyword “bedaquiline” to find all the probable English terms that refer to the drug. The final target terms of BDQ were as follows: “SIRTURO,” “BEDAQUILINE,” “TMC207.” The data was cleaned according to FDA recommendations, and duplicate cases were removed (Shu et al., 2022). BDQ with a reported role coded as “PS” (Primary Suspect) were evaluated for inclusion.

### 2.2 Data mining

In the FAERS database, AEs were encoded and described based on the Preferred Term (PT), which follows a hierarchical classification system with three levels. PT terms were organized according to the System Organ Class (SOC) at the highest level. In the present study, BDQ-related AEs were standardized and classified by SOC and PT of the International Medical Dictionary (MedDRA version 26.1). AE signals are usually detected in the FAERs using disproportionality analysis (DPA), which compares the proportion of AE occurring with specified drugs to identify the signal of AE. The AE signals of BDQ were identified using four different methods (Zhou et al., 2023): Reporting Odds Ratio (ROR), Proportional Reporting Ratio (PRR), Multi-item Gamma Poisson Shrinker (MGPS), and Bayesian Confidence Propagation Neural Network (BCPNN). In order to overcome the limitations of a single algorithm, improve the reliability and accuracy of data mining, and provide more substantial support for the objectives, we have adopted the multi-method strategy. The detailed calculation formula, operation procedure and threshold range were provided in Tables 1, 2. Additionally, we employed logistic regression with R4.2.1 to carry out subgroup analysis on individual data for “hepatotoxicity” and “drug-induced liver injury,” and subsequently, developed a multiple linear regression model.

**TABLE 1:** Contingency table for AE signal detection.

	BDQ related AEs	Non-BDQ related AEs	Total
BDQ	a	b	a+b
Non-BDQ	c	d	c + d
Total	a + c	b + d	a + b + c + d

a, number of reports containing both BDQ and target AEs; b, number of reports containing other AEs of BDQ; c, number of reports containing the target AEs of other drugs; d, number of reports containing other drugs and other AEs.

**TABLE 2:** Four methods, formulas, and thresholds.

Method	Formula	Threshold
ROR	$\text{ROR}=\frac{a/c}{b/d}$	a≥3, lower limit of 95% CI > 1
	$\text{SE}\left(\text{lnROR}\right)=\sqrt{\frac{1}{a}+\frac{1}{b}+\frac{1}{c}+\frac{1}{d}}$ $95\%\text{CI}={\mathrm{e}}^{\ln\left(\text{ROR}\right)\pm 1.96\sqrt{\left(\frac{1}{a}+\frac{1}{b}+\frac{1}{c}+\frac{1}{d}\right)}}$	
PRR	$\text{PRR}=\frac{a/\left(a+b\right)}{c/\left(c+d\right)}$	a≥3,PRR≥2, χ^2^ ≥ 4
	$\chi^{2}=\frac{{\left(\text{ad}‐\text{bc}\right)}^{2}\left(a+b+c+d\right)}{\left(a+b\right)\left(c+d\right)\left(a+c\right)\left(b+d\right)}$	
MGPS	$\text{EBGM}=\frac{a\left(a+b+c+d\right)}{\left(a+c\right)\left(a+b\right)}$	EBGM05 > 2
	$95\%\text{CI}=e^{\ln\left(\text{EBGM}\right)\pm 1.96\sqrt{\frac{1}{a}+\frac{1}{b}+\frac{1}{c}+\frac{1}{d}}}$	
BCPNN	$\text{IC}=\log_{2}\frac{p\left(x,y\right)}{p\left(x\right)p\left(y\right)}=\log_{2}\frac{a\left(a+b+c+\mathrm{d}\right)}{\left(a+b\right)\left(a+c\right)}$	IC025 > 0
	$E\left(\text{IC}\right)=\log_{2}\frac{\left(a+\gamma 11\right)\left(a+b+c+d+\alpha \right)\left(a+b+c+\beta \right)}{\left(a+b+c+d+\gamma \right)\left(a+b+\alpha 1\right)\left(a+c+\beta 1\right)}$	
	$V\left(\text{IC}\right)=\frac{1}{{\left(\ln2\right)}^{2}}\left\{\begin{array}{c} \left[\frac{\left(a+b+c+d\right)‐a+\gamma ‐\gamma 11}{\left(a+\gamma 11\right)\left(1+a+b+c+d+\gamma \right)}\right]+\left[\frac{\left(a+b+c+d\right)‐\left(a+b\right)+\alpha ‐\alpha 1}{\left(a+b+\alpha 1\right)\left(1+a+b+c+d+\alpha \right)}\right]\\ +\left[\frac{\left(a+b+c+d\right)‐\left(a+c\right)+\beta ‐\beta 1}{\left(a+c+\beta 1\right)\left(1+a+b+c+d+\beta \right)}\right] \end{array}\right\}$	
	$\gamma =\gamma 11\frac{\left(a+b+c+d+\alpha \right)\left(a+b+c+d+\beta \right)}{\left(a+b+\alpha 1\right)\left(a+c+\beta 1\right)}$ $\text{IC}‐2\text{SD}=E\left(\text{IC}\right)‐2\sqrt{V\left(\text{IC}\right)}$	
	α1=β1=1,α=β=2,γ11=1	

Abbreviations: ROR, reporting odds ratio; PRR, proportional reporting ratio; MGPS, multi-item gamma Poisson shrinker; BCPNN, bayesian confidence propagation neural network; CI, confidence interval; χ2,chi-squared; IC, information component; IC025, the lower limit of the 95% two-sided CI, of the IC; EBGM, empirical Bayesian geometric mean; EBGM05, the lower 90% one-sided CI of EBGM.

### 2.3 BQD-related DILI network construction

Firstly, we searched the structure and SMILES in PubChem using the keyword “bedaquiline.” The SMILES of BDQ was entered into the SwissTarget Prediction database (Daina et al., 2019), with the species set as “*Homo sapiens*” and a Probability>0 to identify potential effective targets. Simultaneously, the structure of BDQ was input into the PharmMapper database (Liu et al., 2010; Wang et al., 2016; Wang et al., 2017), and targets with a Z score ≥0 were selected. The potential targets collected from both databases were combined, duplicate values were removed, and the obtained targets were standargdized using the UniProt database. Finally, the drug targets related to BDQ were obtained.

The keywords “drug-induced liver injury” were utilized to search for DILI-related targets in the GeneCards and DisGeNET databases. The targets were then sorted based on their Score value, with a higher score indicating greater relevance to the disease. Consequently, a Relevance Score ≥10 was designated as the threshold value for target screening in the GeneCards database, whereas a Score ≥0.1 was employed in the DisGeNET database.

The intersecting genes of BDQ and DILI were obtained by using R software, and duplicates were removed to obtain BDQ-DILI overlaps. The BDQ-DILI intersection targets were uploaded to the STRING online database in order to construct and analyze the protein-protein interaction (PPI) network. The constructed PPI network was imported into Cytoscape 3.8.0 software, and the core targets were selected based on the degree value. Furthermore, the target genes were utilized for Gene Ontology (GO) and Kyoto Encyclopedia of Genes and Genomes (KEGG) enrichment analysis in order to investigate the potential biological pathways involved in BQD-related DILI.

The characterization of drug absorption, distribution, metabolism, excretion, and toxicity is done using ADME/Tox. The SMILES of BDQ was entered into the pkCSM online database (https://biosig.lab.uq.edu.au/pkcsm/prediction) in order to predict its pharmacokinetic properties.

## 3 Results

### 3.1 Basic information on BDQ-related AEs

From October 2012 to December 2023, a total of 16,064,053 case reports were selected in this study based on the search results. After data cleaning, 2017 adverse events related to BDQ were screened for in-depth analysis. The basic information on BDQ-AEs was shown in Table 3. Male users accounted for 52.0% of the recorded BDQ-related AEs, while females accounted for 38.5%. Approximately 20% of AE reports did not include age information. Reports with clear age data showed that the most common age group was 18–64. The main reporting groups included pharmacists (55 cases, 2.7%), physicians (1,550 cases, 76.8%) and consumers (59 cases, 2.9%). The bulk of these reports originated from China, South Africa, and India. AEs leading to hospitalization or prolonged hospitalization were clinically the most common (24.7%), followed by death (21.5%), except for unspecified serious adverse events. The occurrence of AEs was found to be correlated with the duration of medication use. Over the course of the observation period, 342 cases (34.2%) of AEs occurred within 0–30 days after medication, and 142 cases (14.2%) occurred within 31–60 days. Collectively, these AEs accounted for nearly half of the total cases. Our analysis of BDQ adverse events unveiled key insights into its safety and clinical impact. Notably, BDQ was linked to QT interval prolongation and hepatotoxicity, emphasizing vigilant monitoring to prevent severe arrhythmias and liver dysfunction. Comparatively, BDQ showed advantages over other tuberculosis treatments in adverse event severity. Understanding BDQ-induced hepatotoxicity mechanisms, like mitochondrial dysfunction, guides monitoring and treatment decisions. These findings underscored the need for tailored patient care and vigilant monitoring to ensure the safe and effective use of BDQ for multidrug-resistant tuberculosis.

**TABLE 3:** Basic information on AEs related to BQD.

Characteristic	Number of events (%)
Gender	
Female	776 (38.5)
Male	1,049 (52.0)
Unknown	192 (9.5)
Age	
<18	44 (2.2)
18–64	1,449 (71.8)
65–85	144 (7.1)
>85	7 (0.3)
Unknown	373 (18.5)
Weight	
<50 kg	618 (30.6)
50–100 kg	786 (39.0)
>100 kg	6 (0.3)
Unknown	607 (30.1)
Reporter	
Pharmacist	55 (2.7)
Physician	1,550 (76.8)
Consumer	59 (2.9)
Others	232 (11.5)
Unknown	4 (0.2)
Reported Countries	
China	289 (14.3)
South Africa	182 (9.0)
India	178 (8.8)
Uzbekistan	147 (7.3)
Belarus	99 (4.9)
Serious Outcomes	
Death	605 (29.9)
Life-Threatening	126 (6.2)
Hospitalization—Initial or Prolonged	427 (21.1)
Disability	48 (2.3)
AE occurrence time—medication date (days)	
0–30	342 (34.2)
31–60	142 (14.2)
61–90	100 (10.0)
91–180	210 (21.0)
181–360	133 (13.3)
>360	72 (7.2)

### 3.2 BDQ signal mining

**FIGURE 2:**
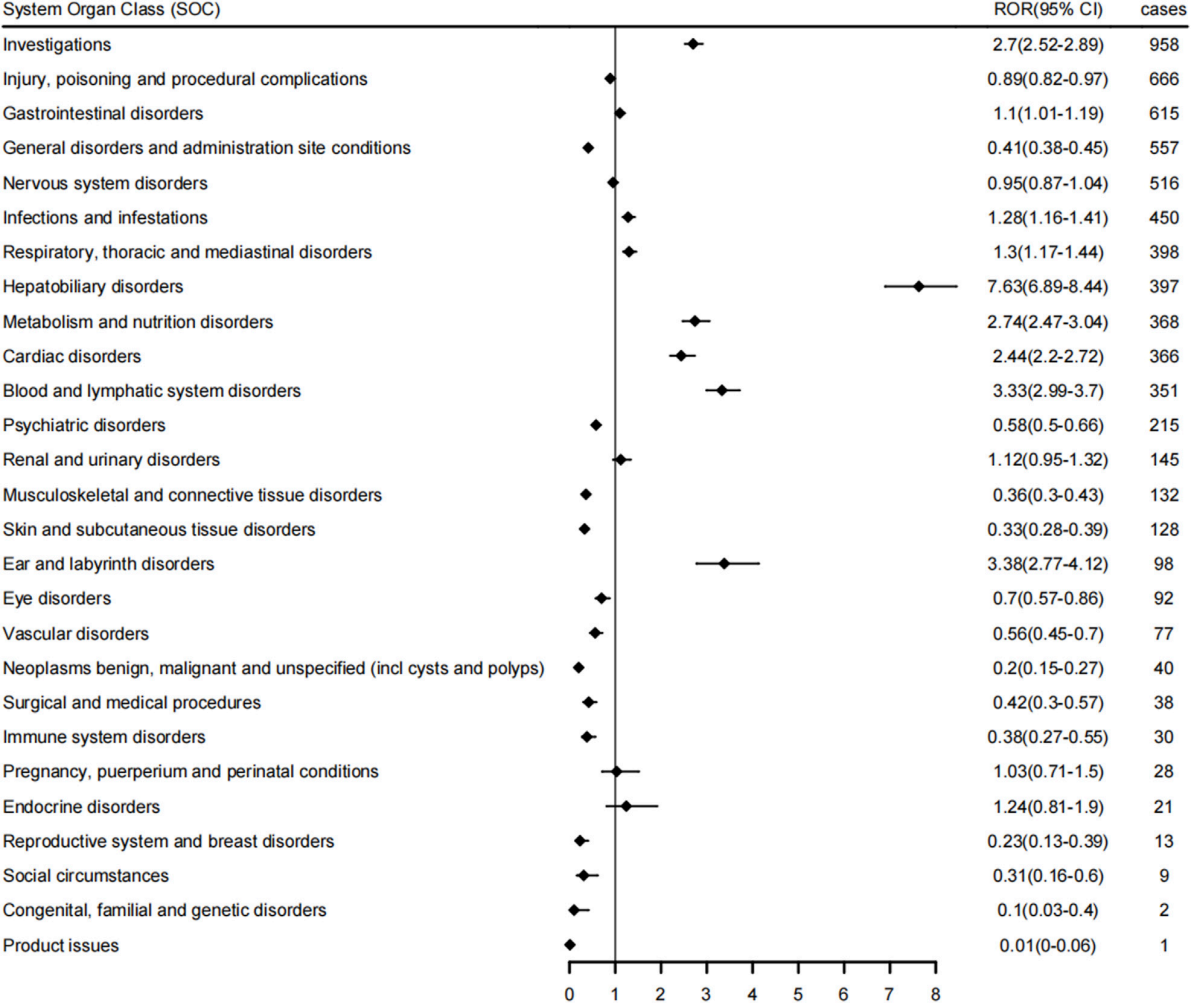
BDQ-related AEs at the SOC level.

The reporting of BDQ-related AEs at the SOC level was shown in Figure 2, revealing that these AEs impacted a total of 27 organ systems. Those most closely linked to BDQ usage were mainly concentrated within the following SOC categories: investigations (*n* = 958, ROR 2.70), gastrointestinal disorders (*n* = 615, ROR 1.10), infections and infestations (*n* = 450, ROR 1.28), respiratory, thoracic and mediastinal disorders (*n* = 398, ROR 1.30), and hepatobiliary disorders (*n* = 397, ROR 7.63). Additionally, we discovered AEs at the SOC level that were not explicitly identified in the BDQ drug instructions, presenting new and valuable AE signals, such as metabolism and nutrition disorders (*n* = 368, ROR 2.74), blood and lymphatic system disorders (*n* = 351, ROR 3.33), and ear and labyrinth disorders (*n* = 98, ROR 3.38).

From the PT-level AEs, a total of 341 PTs that conforming to the four approaches simultaneously. All signals at the PT level were analyzed in the study, and the results of the detection of the top 10 signals with the highest frequency were displayed in Table 4. PTs such as electrocardiogram QT prolonged (*n* = 958, ROR 99.06, PRR 93.83, EBGM 92.42, IC 6.53), was the most frequently reported AE, consistent with the drug label. Furthermore, a number of reports indicated “hepatotoxicity” (*n* = 203, ROR 92.15, PRR 89.39, EBGM 88.12, IC 6.46), suggesting a relatively high frequency of this side effect.

**TABLE 4:** Top ten frequently reported PTs of BDQ.

PT terms	Cases	ROR (95%Cl)	PRR(χ^2^)	EBGM(EBGM05)	IC(IC025)
electrocardiogram QT prolonged	958	99.06 (88.99–110.27)	93.83 (32,398.60)	92.42 (84.49)	6.53 (4.86)
off label use	666	2.92 (2.59–3.29)	2.84 (340.43)	2.84 (2.57)	1.51 (0.16)
anaemia	615	11.01 (9.63–12.59)	10.68 (1949.88)	10.66 (9.53)	3.41 (1.75)
hepatotoxicity	557	92.15 (80.06–106.07)	89.39 (17,493.00)	88.12 (78.33)	6.46 (4.79)
intentional product use issue	516	16.47 (14.20–19.11)	16.06 (2,525.34)	16.02 (14.15)	4.00 (2.34)
vomiting	450	3.47 (2.97–4.05)	3.41 (283.05)	3.41 (3.00)	1.77 (0.1)
neuropathy peripheral	398	14.50 (12.32–17.07)	14.20 (1814.68)	14.17 (12.36)	3.82 (2.16)
tuberculosis	397	78.64 (64.83–95.39)	77.41 (7,896.30)	76.45 (65.05)	6.26 (4.59)
hypokalaemia	368	16.10 (12.80–20.25)	15.93 (1,033.57)	15.89 (13.12)	3.99 (2.32)
aspartate aminotransferase increased	366	15.42 (12.12–19.63)	15.28 (892.49)	15.24 (12.46)	3.93 (2.26)

### 3.3 Subgroup analysis

Data was extracted from PT reports of individuals with PTs named “hepatotoxicity” and “drug-induced liver injury” as target_PT outcome variables for subgroup analysis. Multiple linear regression models were then constructed. Out of the 2017 cases, 238 target_PT individual cases were selected, with 64 cases (26.9%) involving females, 156 cases (65.5%) involving males, and 18 cases with missing data. After adjusting confounders such as age, weight, reporters, and outcome, the results showed a negative correlation between the risk of BDQ-related target_PT and females (OR = 0.4, 95%CI: 0.3–0.6) (Table 5).

**TABLE 5:** Multiple linear regression.

Gender	Model Ⅰ OR (95% CI, P)	Model Ⅱ OR (95% CI, P)
Male	1.0 (Reference)	1.0 (Reference)
Female	0.5 (0.4, 0.7) <0.001	0.4 (0.3, 0.6) <0.001

Outcome variable: PT; Exposure variable: Gender. OR: Odds Ratio. CI: Confidence Interval. Model Ⅰ: Non-adjusted model; Model Ⅱ: adjust model adjust for: age, weight, reporters, outcome.

### 3.4 BQD-related DILI network construction

**FIGURE 3:**
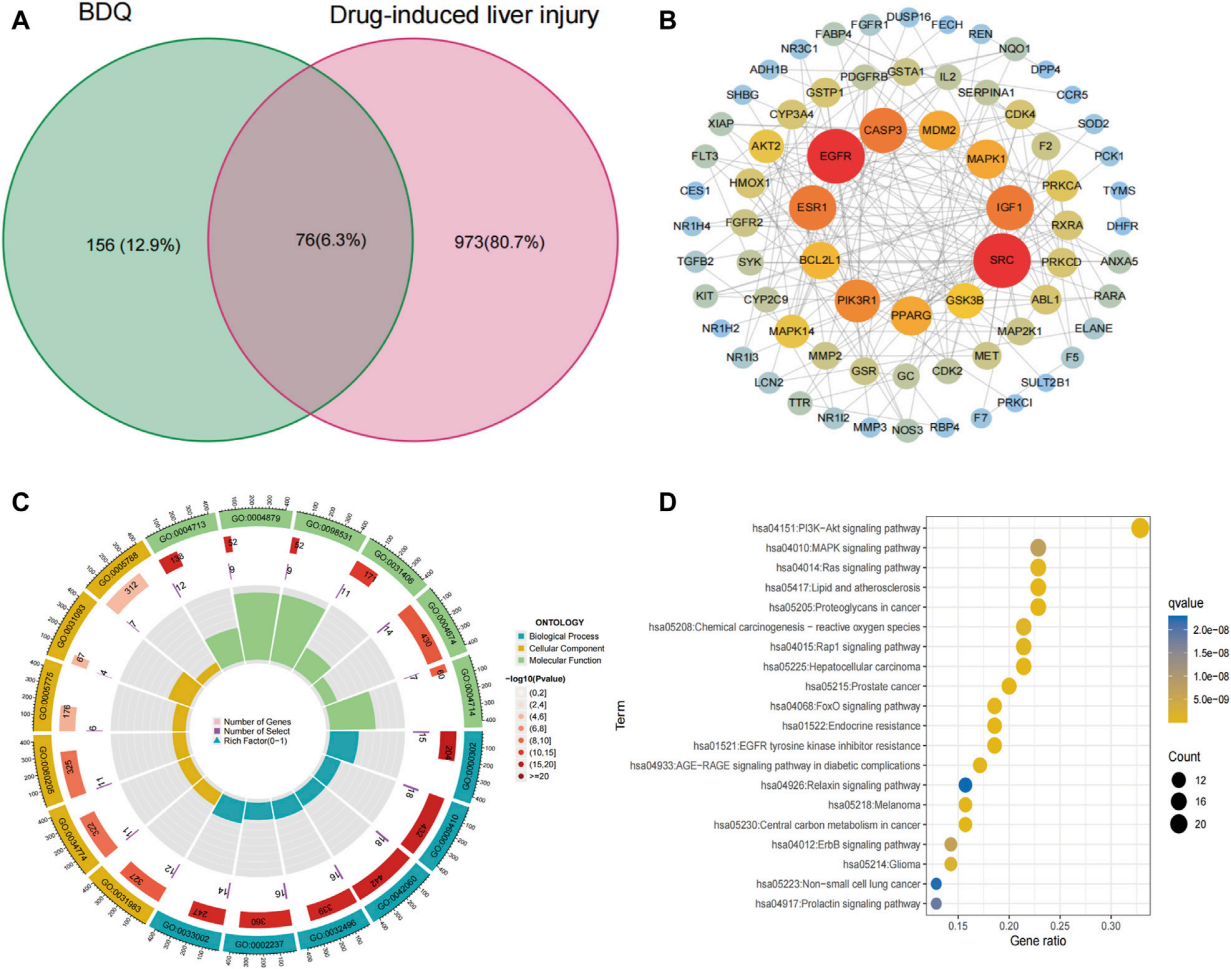
BDQ-DILI network construction. **(A)** Intersection target of BDQ and DILI. **(B)** PPI network. **(C)** GO enrichment analysis. **(D)** KEGG enrichment analysis.

This study identified a total of 232 genes related to BDQ by obtaining 145 drug-related target genes from the PharmMapper database and 100 genes from the SwissTargetPrediction database. Additionally, a total of 1,049 genes related to DILI were obtained by screening 746 target genes from the GeneCards database and 461 targets from the DisGeNET database. By taking the intersection of these two sets of targets, the study identified 76 potential targets for BDQ-associated pharmacologic liver injury Figure 3A. After importing 76 genes into STRING and setting the minimum required interaction score to a high confidence of 0.7, a total of 76 nodes and 198 edges were obtained, with an average node degree of 5.21 and a PPI enrichment *p*-value of less than 1.0 e −16. The BDQ-DILI target PPI network was then analyzed and visualized using Cytoscape (Figure 3B). Nodes with a value of degree greater than 10, such as *SRC, EGFR, ESR1, CASP3, IGF1, PIK3R1, PPARG, MDM2, MAPK1, BCL2L1*, and *GSK3B*, were identified as PPI core genes. GO analysis was conducted on 76 BDQ-DILI genes, resulting in 1443 GO terms that were significantly enriched (adjusted *p*-value <0.05). The top six terms that were significantly enriched in biological processes (BP), cell component (CC), and molecular function (MF) were displayed in Figure 3C. The BP terms (*n* = 1,306) primarily involved response to reactive oxygen species, xenobiotic stimulus, and wound healing, while the CC terms (*n* = 25) encompassed vesicle lumen, secretory granule lumen, and cytoplasmic vesicle lumen. Additionally, the MF terms (*n* = 112) comprised protein tyrosine kinase activity, nuclear receptor activity, and ligand-activated transcription factor activity. The KEGG analysis identified 147 pathways with an adjusted *p*-value of less than 0.05. The top 20 pathways were displayed in Figure 3D, with the PI3K-Akt signaling pathway showing the most significant enrichment among all the pathways. According to statistical analysis, the PI3K-Akt pathway was enriched with 27 BDQ-DILI target genes, and 14 of these targets were found in the top 5 pathways with a high frequency (≥3 times), indicating their significant roles in the enriched pathways. The 14 target genes identified were *PRKCA, MDM2, PDGFRB, AKT2, GSK3B, CDK4, MAPK1, EGFR, FGFR2, MET, IGF1, MAP2K1, PIK3R1*, and *BCL2L1*. Furthermore, the ADME prediction results from the pkCSM online database indicate the presence of “hepatotoxicity” with a predicted value of “yes”.

## 4 Discussion

This study conducted a comprehensive pharmacovigilance analysis of BDQ-related AEs using the FAERS database. From 2012 Q4 to 2023 Q3, we identified 2,017 BDQ-related AEs, with a higher prevalence in males and the most common age group being 18–64 years. The majority of these reports were submitted by physicians, indicating a high level of clinical concern. Hospitalization and death, both serious outcomes, were the results of more than half of these AEs. Early onset of AEs within the first 60 days of medication use was a critical observation, signifying the need for vigilant monitoring during this period.

We identified common adverse events (AEs) including electrocardiogram QT prolongation, hepatotoxicity, vomiting, and others. The association of BDQ with prolonged QT intervals, which was the most frequently reported AE in our study, was consistent with previous research. A retrospective cohort study (Isralls et al., 2021) demonstrated a significant correlation between BDQ use and the prolongation of QT intervals. The significance of this observation lay in the fact that QT prolongation was a known risk factor for the development of potentially fatal arrhythmias, as the statement pointed out (Tisdale et al., 2020). Hepatotoxicity has consistently been identified as a significant adverse effect of BDQ, as evidenced by a previous retrospective study that specifically looked at BDQ-induced liver injury (Kim et al., 2023). Due to the increasing use of BDQ in treating MDR-TB, it was crucial to stress the significance of monitoring liver function in patients undergoing BDQ treatment. Besides the known AEs, our study discovered lesser-known AEs related to BDQ, such as ear and labyrinth disorders. This finding was especially intriguing because it suggested the possibility of BDQ having ototoxic effects, which have not been well reported. Although researchers described one patient who developed tinnitus symptoms after BDQ treatment (Khoza-Shangase and Prodromos, 2021), there are few such reports.

Moreover, this study furthered the understanding of gender disparities in adverse drug reactions. The subgroup analysis indicated a lower risk of BDQ-related liver injury in females (OR = 0.4, 95%CI: 0.3–0.6). Sex-related differences serve as risk factors for the development and severity of liver function impairment of various etiologies (Sayaf et al., 2022). Gender-specific hormonal effects or interaction with signaling molecules can lead to variations in pharmacokinetics between genders, impacting drug effect and safety (Buzzetti et al., 2017). These differences may contribute to the varied AE profiles observed in male and female patients.

In addition, one of the most significant achievements of this study was the construction of the BDQ-related DILI network. From the PPI network, 10 core genes with the highest connectivity degree were selected, out of a total of 76 potential targets identified for BDQ-DILI. The identification of the PI3K/Akt signaling pathway as significantly enriched in BDQ-related DILI was a pivotal finding. The PI3K/Akt pathway (He et al., 2021), famous for its essential role in regulating cell survival, proliferation, and metabolism, has been extensively investigated in diverse diseases, such as cancer (Chawra et al., 2024), inflammation (He et al., 2022), and metabolic disorders (Huang et al., 2018). The coordination of cellular responses to external stimuli, such as drugs or cellular stress, is a critical function of the PI3K/AKT pathway (Guo et al., 2007). This pathway is activated by various growth factors and cytokines, leading to the phosphorylation and activation of Akt (Han et al., 2022). Once activated, Akt supports cell survival by inhibiting apoptosis and promoting cellular growth and proliferation (Izutani et al., 2012). As a result, hepatotoxic drugs may lead to liver injury through the abnormal activation or inhibition of the PI3K/Akt pathway.

Unavoidably, this study was limited in several ways. Firstly, the FAERS database, being a spontaneous reporting system, has inherent limitations such as diverse information sources, redundant data, and missing information. Secondly, the disproportionate analysis used in pharmacovigilance studies does not allow for causal inferences, and further research is needed to confirm these hypotheses. Thirdly, our exploration of the mechanism of BDQ-related liver injury was limited to bioinformatics methods, and further experimental verification is necessary. Despite these limitations, our findings contribute to the exploration of potential adverse events during BDQ treatment, offering a valuable reference for personalized safety measures for patients. Our study shed light on BDQ’s safety profile, crucial for patient care. Specific adverse events like QT interval prolongation and hepatotoxicity necessitated diligent monitoring, including regular ECGs and liver function tests, especially for high-risk patients. Understanding the mechanisms of BDQ-induced liver injury guided clinical decisions, aiding in targeted monitoring and risk mitigation. Comparatively, BDQ exhibited advantages over traditional tuberculosis treatments, especially concerning adverse event severity, guiding treatment selection, particularly for multidrug-resistant tuberculosis (MDR-TB). Tailoring treatment to individual patient profiles was vital, weighing BDQ’s benefits against risks considering factors like comorbidities and medication history. Effective monitoring, including regular follow-ups and patient education, ensures prompt detection and management of adverse events, fostering collaborative patient-provider relationships for optimal care.

## 5 Conclusion

In conclusion, our study utilized signal mining techniques on the FAERS database in order to analyze AE signals connected to BDQ. Apart from the typical side effects like prolonged QT interval, vomiting, and hepatotoxicity outlined in the package insert, it was crucial to pay attention to emerging risks such as ototoxicity. For male patients receiving bedaquiline (BDQ) therapy for tuberculosis, stringent monitoring of liver function was imperative to mitigate the risk of severe hepatotoxicity and ensure medication safety. Moreover, investigating the underlying biological mechanisms of BDQ-induced liver injury, particularly involving the PI3K/Akt pathway, could offer valuable insights for future research endeavors. Healthcare providers played a pivotal role in augmenting patient safety during bedaquiline prescription by conducting comprehensive pre-treatment evaluations, facilitating informed consent and patient education, instituting regular surveillance for adverse events, tailoring treatment approaches to individual needs, and promptly addressing any identified adverse reactions through appropriate dosage adjustments or supportive interventions. These multifaceted strategies collectively safeguard patient wellbeing and optimize treatment outcomes in BDQ therapy.

## Data Availability

The original contributions presented in the study are included in the article/Supplementary material, further inquiries can be directed to the corresponding authors.
